# Role of Proton-Coupled Monocarboxylate Transporters in Cancer: From Metabolic Crosstalk to Therapeutic Potential

**DOI:** 10.3389/fcell.2020.00651

**Published:** 2020-07-17

**Authors:** Xiangyu Sun, Mozhi Wang, Mengshen Wang, Litong Yao, Xinyan Li, Haoran Dong, Meng Li, Tie Sun, Xing Liu, Yang Liu, Yingying Xu

**Affiliations:** ^1^Department of Breast Surgery, The First Affiliated Hospital of China Medical University, Shenyang, China; ^2^The Second Affiliated Hospital of China Medical University, Shenyang, China

**Keywords:** tumor microenvironment, metabolic networks and pathways, monocarboxylic acid transporters, lactic acid, glycolysis

## Abstract

Proton-coupled monocarboxylate transporters (MCTs), representing the first four isoforms of the *SLC16A* gene family, mainly participate in the transport of lactate, pyruvate, and other monocarboxylates. Cancer cells exhibit a metabolic shift from oxidative metabolism to an enhanced glycolytic phenotype, leading to a higher production of lactate in the cytoplasm. Excessive accumulation of lactate threatens the survival of cancer cells, and the overexpression of proton-coupled MCTs observed in multiple types of cancer facilitates enhanced export of lactate from highly glycolytic cancer cells. Proton-coupled MCTs not only play critical roles in the metabolic symbiosis between hypoxic and normoxic cancer cells within tumors but also mediate metabolic interaction between cancer cells and cancer-associated stromal cells. Of the four proton-coupled MCTs, MCT1 and MCT4 are the predominantly expressed isoforms in cancer and have been identified as potential therapeutic targets in cancer. Therefore, in this review, we primarily focus on the roles of MCT1 and MCT4 in the metabolic reprogramming of cancer cells under hypoxic and nutrient-deprived conditions. Additionally, we discuss how MCT1 and MCT4 serve as metabolic links between cancer cells and cancer-associated stromal cells via transport of crucial monocarboxylates, as well as present emerging opportunities and challenges in targeting MCT1 and MCT4 for cancer treatment.

## Introduction

Monocarboxylate transporters (MCTs) belong to the *SLC16A* gene family and comprise 14 members. Of these identified 14 members, the proton-coupled isoforms MCT1–4 are critical in the metabolic process due to their roles in the transport of monocarboxylates such as lactate, pyruvate, and ketoacids (Halestrap and Price, 1999; Halestrap and Meredith, 2004). The function of proton-coupled MCTs as transporters of monocarboxylates is crucial for the metabolic rewiring of tumor cells and stromal cells (Martinez-Outschoorn U. et al., 2014; Pucino et al., 2018). Cancer cells display a metabolic shift from oxidative metabolism to glycolysis to rapidly produce ATP and lactate in the cytoplasm (Koppenol et al., 2011). The lactate export mediated by proton-coupled MCTs is essential for the survival of cancer cells (Ganapathy et al., 2009; Draoui and Feron, 2011). Moreover, cancer cells exist within a complex microenvironment, surrounded by stromal cells primarily including immune cells, endothelial cells, and fibroblasts. The metabolic interplay between tumor cells and these stromal cells provides advantages for tumor proliferation and progression. MCT1 and MCT4 play the most dominant role in the transport of monocarboxylates, and they have been found to be significantly upregulated and associated with poor prognosis in multiple malignant tumors including peritoneal carcinomatosis, prostate cancer, lymphoma, and oral cavity cancer (Pértega-Gomes et al., 2011; Simões-Sousa et al., 2016; Kim et al., 2018; Afonso et al., 2019). Compared to MCT1 and MCT4, MCT2 and MCT3 have been less studied in cancer. Therefore, this review explores the metabolic crosstalk and therapeutic implications of proton-coupled MCTs with a primary focus on the current roles of MCT1 and MCT4 as targets in cancer.

## The Metabolic Roles of MCT1 and MCT4 in Cancer Cells

MCT1 and MCT4 are crucial players in the process of lactate exchange within tumors. The two isoforms differ in multiple aspects including biochemical properties and tissue distribution (Halestrap and Meredith, 2004; Pérez-Escuredo et al., 2016). MCT1, which exhibits a high affinity for lactate, preferentially facilitates lactate uptake to fuel oxidative phosphorylation (OXPHOS) and is also involved in lactate efflux from cancer cells. MCT4 is a low-affinity, lactate-preferring transporter, adapted to export lactate from glycolytic cancer cells (Dimmer et al., 2000; Halestrap and Meredith, 2004). It should be noted that both normoxic and hypoxic cancer cells co-exist within the tumors, with a lactate shuttling commonly observed between hypoxic and normoxic sites. Hypoxic tumor cells depend on glucose as the main fuel source to satisfy their energy demand, and the high levels of lactate produced are subsequently transported out of the cytoplasm primarily via MCT4. Concurrently, tumor cell-derived lactate can enter the neighboring normoxic cancer cells via MCT1 for OXPHOS, thus sparing glucose for glycolytic cancer cells (Sonveaux et al., 2008; Doherty and Cleveland, 2013). This form of metabolic symbiosis illustrates how the apparent waste product from hypoxic tumor cells may be exploited by oxidative tumor cells to sustain their energy production under nutrient-deprived condition. The relative contributions of glucose and lactate to the tricarboxylic acid (TCA) cycle of non-small cell lung carcinoma cells have been compared. Infusing tumors with 13C-labeled lactate resulted in higher amounts of labeled metabolites TCA cycle metabolites compared to infusion with 13C-labeled glucose (Faubert et al., 2017). These findings highlight that the lactate transport between hypoxic and normoxic tumor cells mediated by MCT1/4 may be crucial for energy production, tumor proliferation and invasion.

An additional role of MCTs is their capability to transport pyruvate across the plasma membrane. Pyruvate is the end-product of glycolysis and a turning point for production of glucose, lactate, fatty acids, and amino acids. As a significant substrate for the TCA cycle, cytoplasmic pyruvate can be transported into the mitochondria to further undergo oxidative metabolism to produce ATP and provide important intermediates. MCT4 shows a high affinity for pyruvate, which may prevent pyruvate efflux that is essential for maintaining of a high glycolytic flux (Halestrap, 2013). MCT1, with a lower affinity for pyruvate, is responsible for the bidirectional transport of pyruvate (Halestrap, 2013). Hong et al. (2016) proposed that MCT1 expression is elevated in glycolytic cancers to enhance pyruvate export to impair mitochondrial OXPHOS. MCT1 inhibition blocks pyruvate export without glycolysis impairment in glycolytic breast tumor cells co-expressing MCT1 and MCT4, which promotes mitochondrial oxidative metabolism and suspends tumor growth (Hong et al., 2016). However, Diers et al. (2012) found that MCT1 upregulation may enhance pyruvate uptake in breast cancer cells, fueling OXPHOS and proliferative potential. MCT inhibition impaired mitochondrial respiration and decreased cell growth through inhibition of cellular pyruvate uptake (Diers et al., 2012). Although the precise roles of MCT1/4 in the pyruvate transport of cancer cells need further investigation, these conflicting results proposed alternative molecular mechanisms for the action of MCT inhibitors in addition to their effects on lactate transport.

## How MCT1 and MCT4 Link Cancer Cells and the Tumor Microenvironment

**FIGURE 1:**
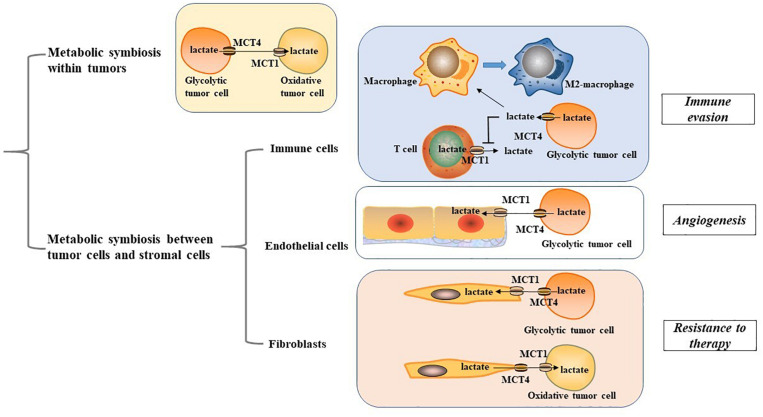
Metabolic symbiosis within tumor microenvironment. MCT1, monocarboxylate transporter 1; MCT4, monocarboxylate transporter 4; M2 macrophage, alternatively activated macrophage.

MCT1 and MCT4 are not only essential for metabolic symbiosis between glycolytic and oxidative cancer cells; they also mediate metabolic rewiring within the tumor microenvironment (TME). MCT1/4-mediated metabolic interplay between cancer cells and stromal cells may be critical for tumor immune response, angiogenesis, and therapy resistance (Figure 1). Thus, targeting the metabolic interplay within the TME is indispensable for developing new interventions for cancer management.

### Immune Cells

Cancer cells display metabolic crosstalk with immune cells using a variety of mechanisms. Studies have shown that high levels of lactate produced by glycolytic tumor cells can lead to immune evasion (Bohn et al., 2018; Sekine et al., 2018). Macrophages, a key representative of myeloid lineages possess two polarized types of the classically activated (M1) macrophage phenotype and the alternatively activated (M2) macrophage phenotype (Biswas and Mantovani, 2010). MCT1/4-mediated lactate secretion from cancer cells plays an important role in mediating macrophage polarization to the M2-like state, which presents with immunosuppressive properties (Colegio et al., 2014; Ohashi et al., 2017; Mu et al., 2018; Liu et al., 2019; Stone et al., 2019). In gastric cancer, the lactate-MCT-hypoxic inducible factor-1α (HIF-1α) axis has been identified as a crucial signaling axis that connects metabolic rewiring and immune evasion. MCT1 inhibition or knockdown of HIF-1α can remarkably abrogate the expression of key M2 marker CD163 and arginase 1 in macrophages (Zhang and Li, 2020). Activation of nuclear factor E2-related factor-2, a transcription factor for regulating oxidative stress response, can induce MCT1 upregulation, fuel lactate uptake of premalignant colonic epithelial cells exposed to inflammatory M1 macrophages and shift colonic epithelial cells toward a reversed Warburg metabolism, favoring malignant transformation of the colonic epithelium (Diehl et al., 2018). This adds new insights for the impact of MCT1/4-mediated lactate transport on macrophage functionality. Nevertheless, the carcinogenic role on MCT 1/4 in macrophages may not be restricted to their effect on lactate transport. In glioblastoma, transport of branched-chain ketoacids (BCKAs), metabolites of branched-chain amino acid catabolism, is also mediated by MCT1. Tumor-excreted BCKAs can be taken up and re-aminated to branched-chain amino acids in macrophages. Exposure to BCKAs abrogates the phagocytic activity of macrophages, and the anti-proliferative effects of MCT1 silencing may also be involved with impaired excretion of BCKAs (Silva et al., 2017). These findings imply a significant role of MCT1/4 in mediating the metabolic crosstalk between tumor cells and macrophages to provide potential metabolic vulnerabilities for cancer management.

T cells have a decisive protective role in host defenses against cancer, and therapeutic strategies targeting T cell immunometabolism have developed quickly in the past years (Jiang and Yan, 2016; Kishton et al., 2017). It is well established that T cells undergo metabolic adaption for the bioenergetic needs of their immune response. During activation, T cells rely on their enhanced glycolytic phenotype to support their growth and effector functions (Thommen and Schumacher, 2018). However, high lactate concentration in the TME blocks lactate export from T cells, thereby disturbing their metabolism and function (Brand et al., 2016). For instance, cytolytic CD8+ T lymphocytes have been found to rely on MCT1-mediated lactate export to sustain their cytokine production and cytotoxic activity (Fischer et al., 2007). Thus, targeting this metabolic pathway in tumors is a promising strategy to enhance tumor immunogenicity. It has been found that silencing MCT1 and MCT4 can suppress lactate secretion, restore T cell-induced immune function, and boost response to immune checkpoint inhibitors in melanoma patients (Renner et al., 2019). Given that immune checkpoint inhibitors function to activate the effective immune response of T cells, MCT1/4 provide new insights for cancer immunotherapy.

### Endothelial Cells

The Warburg effect, accompanied with high MCT1/4 expression in the tumor-endothelial cells (EC) micro-environment contributes significantly to angiogenic capacity and cancer progression (Rohlenova et al., 2018; Guo et al., 2019). Glycolytic cancer cells primarily depend on MCT4 to release lactate into the TME, ECs take up lactate in a MCT1-mediated manner, similar to oxidative cancer cells. MCT1/4-mediated lactate transport has been shown to serve as a crucial pro-angiogenic factor to mediate tumor-EC metabolic rewiring, angiogenic activity, and tumor progression in multiple cancers, including glioblastoma, renal cancer, colorectal, and breast cancer, which may rely on activation of oncogenic signaling including nuclear factor-κB (NF-κB) and HIF-1α (Miranda-Gonçalves et al., 2017; Rohlenova et al., 2018; Guo et al., 2019). In tumor ECs, lactate activates the vascular endothelial growth factor signaling pathway to promote lactate-mediated proangiogenic activity, which may act through a poly-ADP ribosylation-dependent mechanism (Kumar et al., 2007). Upon entry into the ECs, lactate can be oxidized by lactate dehydrogenase to produce pyruvate that can subsequently interact with prolylhydroxylases and inhibit prolylhydroxylases activity. This promotes an autocrine NF-κB/IL-8 pathway to drive angiogenic activity and tumor progression (Vegran et al., 2011). The pro-angiogenic properties of the tumor-EC network suggest that MCT1/4 have valuable implications for new therapeutic concepts targeting tumor angiogenesis.

### Fibroblasts

In tumor-stroma contact models, a symbiotic relationship termed as the reverse Warburg effect has been established in which stromal cells are induced by oxidative cancer cells to undergo a glycolytic switch and MCT4 upregulation, and metabolites including lactate and pyruvate are imported into the cancer cells for OXPHOS in a MCT1-dependent manner. This metabolic compartmentalization creates a nutrient-rich microenvironment to produce mitochondrial fuels and enables oxidative cancer cells to satisfy their metabolic demand (Koukourakis et al., 2006; Martinez-Outschoorn U.E. et al., 2014; Whitaker-Menezes et al., 2014; Sakamoto et al., 2019). The catabolic phenotypes observed in cancer-associated fibroblasts (CAFs) are driven by multiple cell signaling pathways including loss of caveolin-1 and the activation of HIF-1α and NF-κB signaling, which may serve as metabolic vulnerabilities in the metabolic rewiring of the tumor-CAF network (Pavlides et al., 2009; Bonuccelli et al., 2010; Wilde et al., 2017). In a prostate cancer cell model, tumor-CAF contact triggers a sirtuin 3 -mediated regulation of HIF1 stabilization and glycolytic phenotype to upregulate MCT4 and enhance lactate secretion from CAFs, while prostate cancer cells increase lactate uptake mediated by MCT1 (Fiaschi et al., 2012). Consistently, prostate cancer cells have been shown to undergo sirtuin 1-dependent PGC-1α activation to enhance mitochondrial OXPHOS (Ippolito et al., 2019). Therefore, the reverse Warburg effect mediated by MCT1/4 leads to an enhanced malignant phenotype, making it a crucial therapeutic target.

However, opposite situation exist wherein stromal cells metabolically use lactate and other monocarboxylates exported by highly glycolytic cancer cells for their own energetic requirements. This metabolic interplay not only spares glucose for adjacent glycolytic tumor cells, but it also enables CAFs to provide metabolites of lactate oxidation, such as pyruvate, to meet the energy needs of tumor cells (Koukourakis et al., 2006, 2017; Patel et al., 2017). It has been shown that tyrosine kinase inhibitors-resistant cancer cells exhibit an enhanced glycolytic phenotype and lactate production, while CAFs increase their lactate uptake via MCT1. This further induces upregulation of HGF transcriptional level in CAFs through an NF-kB-dependent manner, which leads to drug resistance to tyrosine kinase inhibitors (Apicella et al., 2018). These emerging opposite situations indicate the existence of tumor metabolic heterogeneity, highlighting the need for further investigations into the metabolic interplay between tumor cells and CAFs.

## Implications for Cancer Treatment

Based on its role in facilitating tumor progression, blocking MCT1/4-mediated monocarboxylate transport may provide novel insights for cancer therapy. Several MCT1 inhibitors have been described, especially AZD3965 and its analog AR-C155858. AZD3965 is a specific MCT1/2 inhibitor that is currently being evaluated in a phase 1 clinical trial^1^ in solid tumors (NCT01791595). AZD3965 has shown promising anti-tumor effects in treating MCT1-overexpressing models of diverse malignancies including Burkitt lymphoma, diffuse large B-cell lymphoma, gastric cancer, and small cell lung cancer (Bola et al., 2014; Polanski et al., 2014; Noble et al., 2017). Evidence indicates that AZD3965 and its analog AR-C155858 may not be effective. *In vivo* AR-C155858 treatment was shown to be ineffective in the murine 4T1 xenograft breast tumor model, which may relate to the immune status of the preclinical xenograft model (Guan et al., 2018). Another disadvantage of MCT1-selective inhibition is that it is ineffective when MCT4 is expressed due to the compensatory effect of MCT4 for MCT1 activity (Fiaschi et al., 2012). However, MCT4 inhibitors are still in the discovery phase. Developing drugs that co-inhibit MCT1 and MCT4 may be more effective in blocking lactate secretion and tumor growth. However, inhibition of lactate uptake via MCT1/4 inhibitor may drive glucose influx to mitochondrial metabolism to maintain tumor cell survival (Beloueche-Babari et al., 2017; Corbet et al., 2018). Therefore, co-administration of MCT1/4 inhibitors and mitochondrial-targeted therapy, such as the mitochondrial complex I inhibitor metformin or mitochondrial pyruvate carrier inhibitors, may counteract the elevated mitochondrial metabolism (Beloueche-Babari et al., 2017; Benjamin et al., 2018). It is noteworthy that MCT1 may mediate tumor progression beyond its role as a lactate transporter. For instance, MCT1 has been found to activate the transcription factor NF-κB to facilitate tumor cell migration independently of its transporter activity (Payen et al., 2017). This suggests that coordination of MCT1 inhibitors and other therapeutic agents to block tumor development may be a key point for future pharmacological strategies.

CD147 is a transmembrane glycoprotein that whose overexpression significantly correlates with poor prognosis in multiple malignancies (Nabeshima et al., 2006; Iacono et al., 2007; Pinheiro et al., 2009; Zhong et al., 2012; Huang et al., 2018; Landras et al., 2019). Importantly, CD147 forms complex with MCT1/4, which is necessary for maintaining MCT1/4 cell surface expression and activity (Kirk et al., 2000; Gallagher et al., 2007; Eichner et al., 2016). Therefore, the major pro-tumoral action of CD147 was shown to involve a metabolic modification of the TME through its interaction with MCT1 and MCT4 (Le Floch et al., 2011; Marchiq et al., 2015; Updegraff et al., 2018). Currently, potential CD147 inhibitors including p-chloromercuribenzene sulfonate, which disrupts CD147-MCT1/4 interaction, and AC-73, which targets CD147 dimeric interface, have been proposed (Wilson et al., 2005; Spinello et al., 2019). These studies illustrate that CD147 has therapeutic implications for cancer treatment in addition to directly target proton-coupled MCTs.

## Conclusion

Proton-coupled MCTs, especially MCT1 and MCT4, are emerging as promising therapeutic targets for cancer treatment. Indeed, MCT1/4-mediated transport of metabolites such as lactate and pyruvate not only plays a decisive role in metabolic symbiosis between hypoxic and normoxic cancer cells within tumors and also links crosstalk between cancer cells and stromal cells including immune cells, endothelial cells and fibroblasts. These metabolic interplays mediated by MCT1 and MCT4 necessitate further exploration in the clinical settings. MCT1 inhibitors are currently being tested in the clinical trial, and potential combination with other agents may provide new prospects for cancer management. CD147, which is necessary for MCT1 and MCT4 activity, also serves as a therapeutic target to block MCT1/4-mediated transport of crucial metabolites to impair cancer progression. Unfortunately, our understanding of the effects of MCT1/4 are still limited and most studies are based on *in vitro* and preclinical data. Thus, a better understanding of the role of MCT1 and MCT4 in metabolic reprogramming and cancer development is required for novel therapeutic strategies.

## Author Contributions

XS wrote and elaborated the figures. MoW, MeW, LY, XLi, HD, ML, TS, and XLiu wrote and reviewed the manuscript. YL and YX wrote and reviewed the final version. All authors contributed to the article and approved the submitted version.

## Conflict of Interest

The authors declare that the research was conducted in the absence of any commercial or financial relationships that could be construed as a potential conflict of interest.

## Footnotes

1 www.clinicaltrials.gov
